# A novel strategy of using ultraviolet a light emitting diodes (UVA-LED) irradiation extends the shelf-life and enhances the antioxidant property of minimally processed pakchoi (*Brassica rapa* subsp. *chinensis*)

**DOI:** 10.1016/j.fochx.2025.102826

**Published:** 2025-07-22

**Authors:** Yamin Fan, Wen Huang, Han Gao, Shun Zhang, Xiaoyan Zhang, Jianshe Zhao, Lijuan Zhan

**Affiliations:** a College of Food Science and Technology; Henan Agricultural University, Zhengzhou 450002, China; b Zhengzhou Academy of Agricultural Science and Technology, Zhengzhou 450015, China; c Henan Zhongyuan Organic Agriculture Institute Co.Ltd., Zhengzhou 450000, China

**Keywords:** UV irradiation, Postharvest quality, Antioxidant capacity, Shelf-life, Leafy vegetable

## Abstract

Ultraviolet A light-emitting diode (UVA-LED) is an emerging non-chemical technology with potential of enhancing postharvest quality of fresh produce. This study compared effects of UVA-LED irradiation (0-control, 4, 8, 12, 16, 20 J/cm^2^) on senescence and shelf-life of minimally processed pakchoi (MPP). An 8 J/cm^2^ dose effectively preserved chlorophyll and color, extending 2 days shelf-life. Further analysis revealed that UVA-LED (8 J/cm^2^) increased phenolic acids (+ 18.21 %) and flavonoids (+ 57.50 %) at d5 by activating enzymes (phenylalanine ammonia-lyase-(PAL), cinnamate-4-hydroxylase-(C4H), and 4-coumarate: coenzyme A ligase-(4CL)), and upregulating corresponding genes. Moreover, UVA-LED enhanced 30 % - 40 % total antioxidant capacity (TAC) at d5, associated with elevated levels of both enzymatic (superoxide dismutase (SOD, +22.75 %), catalase (CAT, +68.00 %), and peroxidase (POD, +45.81 %), and non-enzymatic (total phenolics (1.21-fold), total ascorbic acid (1.90-fold)) antioxidants. However, UVA-LED accelerates fresh weight loss. Conclusively, proper dose UVA-LED irradiation presents an innovative strategy to preserve vegetables postharvest quality.

## Highlights

• UVA-LED affects pakchoi postharvest quality, but highly dose-dependent.
• An 8 J/cm^2^ dose is effective to preserve quality and extend shelf-life of pakchoi.
• UVA-LED enhances antioxidant property of pakchoi by inducing antioxidants.
• UVA-LED accumulates phenolics via key enzyme activation and genes expression.
• UVA-LED accelerated fresh weight loss of postharvest pakchoi during storage.

## Graphical abstract

**Figure:**
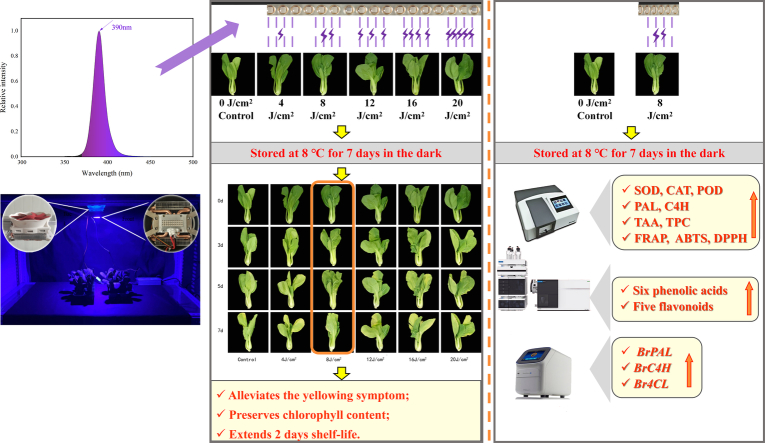

## 1 Introduction

Pakchoi (*Brassica rapa* subsp. *chinensis*), belonging to *Brassica* species, is an excellent source of bioactive compounds including carotenoids, ascorbic acid, phenolic acids, and flavonoids, in addition to dietary fiber and mineral elements (Li et al., 2018). These compounds are well known for their antioxidant and anti-inflammatory properties, contributing to various health-promoting effects (Sorrenti et al., 2023). After harvest, pakchoi is typically marketed as a 100 % edible canopy, either packaged or unpackaged, known as minimally processed pakchoi (MPP). MPP is very susceptible to water loss and yellowing resulting from the large leaf surface area and chlorophyll deterioration after harvest, which significantly reduces its nutritional quality and shelf-life. At ambient room temperature, MPP has only approx. 1–2 days shelf-life without any treatment because of the quick wilting and yellowing, seriously limiting its transportation and sale during supply chain. Traditional postharvest chemical treatment, such as hydrogen peroxide and chlorine-containing solution, are not applicable on minimally processed leafy vegetable due to both undesirable odor and the growing public concern on food safety, although they are effective in disinfecting and sterilizing food material (Yuan et al., 2021). Therefore, to fully meet the needs of modern food processors and consumers for low cost, safe, and nutritious foods, a feasible innovative preservation technology is imperative to extend the shelf-life and retain nutritional quality of postharvest pakchoi.

In past few years, Ultraviolet light emitting diode (UV-LED) irradiation has emerged as a novel, non-thermal, non-chemical, and residue-free promising physical technology to gain growing interest in food preservation (Salazar et al., 2022). Compared to conventional mercury UV lamp, UV-LED has the advantages of higher energy efficiency, lower heat generation, environmentally friendly (no mercury), and diverse wavelengths (D'Souza et al., 2015). Typically, the UV spectrum is divided into UVA (320–400 nm), UVB (280–320 nm), and UVC (200–280 nm), all of which potentially can be used for the treatment of food products (Salazar et al., 2022). In comparison to UVB and UVC, UVA represents the primary type of natural UV irradiation available to plants, as UVC is completely and UVB is mostly absorbed by the ozone layer of the atmosphere (Lee et al., 2019). Moreover, UVA is more moderate and causes less fatal damage to plant tissue because of longer wavelength and lower energy, making it safer and practical for application in controlled environment agriculture (CEA) (Choi et al., 2022). For these reasons, UVA-LEDs are typically applied before harvest to stimulate the growth and improve the content of photosynthetic pigments and antioxidant compounds of leafy vegetables. For instance, preharvest supplementary of UVA-LED could stimulate biomass production and secondary metabolite accumulation of lettuce (Hooks et al., 2022), improve the growth and bioactive compounds of kale (Lee et al., 2019), and enhance phytochemicals and antioxidant properties of pakchoi microgreens (Brazaityte et al., 2015). These studies have strongly suggested a possibility to use UVA-LED as an applicable preharvest strategy to increase the content of various phytochemicals and antioxidant capacity of leafy vegetables.

To date, however, an overview of the literature revealed that the researches on postharvest application of UVA-LED for quality preservation of fresh fruit and vegetables are highly limited. The few available studies have showed that the application of UVA irradiation is effective for vegetable surface sterilization (365 nm, 675 J/m^2^), enhancing the accumulation of bioactive compounds in *Scutellaria baicalensis* root (4 W/m^2^ for 7 days, 8 h per day), and improving the antioxidant activity of tomato fruits (365 nm, 0.28 W/m^2^ for 360 min) (Aihara et al., 2014; Dyshlyuk et al., 2020; Miao et al., 2022). A conflict result was also reported that post-harvest use of UVA lamp (366 nm, 1 kJ/m^2^ dose) alone showed no significant improvement in nutritional quality of tomato fruit (Baenas et al., 2021). However, the majority of existing researches has focused on the effect of conventional UVA lamps on the postharvest fruits or roots, with a notable absence of studies on leafy vegetables. It is worth noting that, differing from fruit and root, the vegetable leaves still retain the ability to perceive light stimuli even after harvest and respond in ways that affect cellular metabolism (Zhan et al., 2020). This continued light responsiveness provides a unique opportunity to regulate postharvest metabolism in a way that promotes overall quality of product. Taking advantage of this active responsiveness may represent a promising strategy for enhancing the postharvest quality and shelf-life of fresh leafy vegetables.

This study investigated the effect of UVA-LED on postharvest shelf-life and antioxidant property of MPP by carrying out two experiments. The first trial aimed to identify the suitable UVA-LED irradiation dose by comparing the effects of various doses of 0 (control), 4, 8, 12, 16, and 20 J/cm^2^ on senescence (yellowing symptom), chlorophyll content, and shelf - life of MPP during storage. The second experiment investigated the impact of the selected UVA-LED dose on antioxidant property of MPP, with a particular focus on the phenolic metabolism (total phenolic compounds – TPC, phenolic acids - PAs, flavonoids compounds, enzymes activity and related gene expression), antioxidant enzyme activity (superoxide dismutase - SOD, catalase - CAT, peroxidase - POD), ascorbic acid content, and total antioxidant capacity (TAC). Additionally, other physiological parameters, including the headspace gas content (O_2_ and CO_2_), fresh weight loss (FWL), and dry matter (DM) content, were also assayed.

## 2 Material and methods

### 2.1 Material preparation and irradiation treatment

**Fig. 1:**
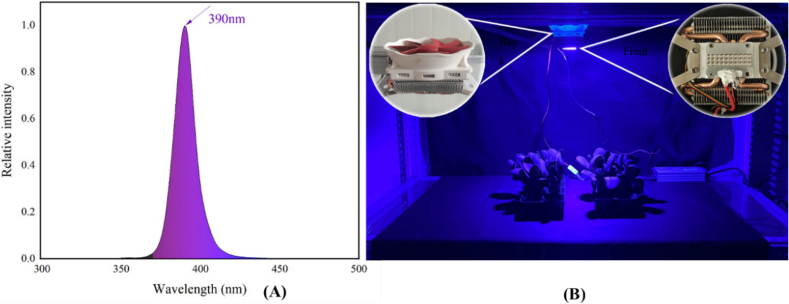
The UVA-LED wavelength peak at 390 nm (A) and irradiation treatment (B).

Fresh pakchoi was obtained from local market in Zhengzhou, China, and immediately transported to the laboratory. The outer leaves with any mechanical injury or visible disease symptom were manually removed. Plant canopies with 3–4 mature leaves, selected based on their uniform size and color, were then washed with cool water (12 ± 1 °C). The excess surface water was gently removed using absorbent paper. About 150 g pakchoi for each sample was arranged upright in the plastic tray (25.5 cm * 12.8 cm * 5.5 cm). In the first experiment, total of 72 samples (72 samples = 6 irradiation doses ×3 replicates ×4 sampling days) were randomly divided into 6 batches equally. Each batch was irradiated using a customized UVA-LED with a wavelength peak at 390 nm (Hubei DUVTek Co., Ltd., Hubei, China) (Fig. 1). The irradiation intensity reaching the sample surface were measured at 12 mW/cm^2^ using plant lighting analyzer (OHSP-350P, Zhejiang Hopoo Light & Color Technology Co., Ltd., Hangzhou, China). The corresponding irradiation dose of 0 (control), 4, 8, 12, 16, and 20 J/cm^2^ was obtained by multiplying irradiation intensity by irradiation time. After irradiation, the trays were packaged with plastic film (Changjiang Hengjiu Plastic Co., Ltd., Hangzhou, China) and sealed with the heat sealer (T-300 treadle sealing machine, Shanghai Riou Industrial Co., Ltd., China). The packaged samples were stored in customized storage room (Zheng Zhou Xing Rui Refrigeration Equipment Co., Ltd., Zhengzhou, China) at 8 ± 2 °C in darkness. The relative humidity was 80 ± 5 % in the storage room. The yellowing symptom, chlorophylls content, and shelf-life were detected during storage.

Basing on the first trial result, the second experiment was carried out to further evaluate the effect of optimal irradiation dose (8 J/cm^2^) on antioxidant property of MPP. In this case, total of 24 samples, that was, 24 samples = 2 doses (0-control and 8 J/cm^2^) × 3 replicates ×4 sampling days, were prepared. These samples were irradiated, packed, heat-sealed, and stored as description in first experiment.

At sampling day, for analysis of chlorophyll, TPC, ascorbic acid (AA), TAC, and enzymes activities, the plant tissue was wrapped with aluminum foil and frozen with liquid nitrogen immediately and then stored in - 60 °C refrigerator until analysis. For PAs and flavonoid compounds assay, the plant tissue was frozen drying for 48 h in vacuum freeze dryer (LGJ-18S, Beijing Songyuan Huaxing Technology Development Co., Ltd., Beijing, China). After that, the leaves were ground into powder and stored in - 60 °C refrigerator for further assay.

### 2.2 Senescence and chlorophyll *a*ssay

On the day of sampling, the senescence (yellowing symptoms) was monitored and photographed. For chlorophyll (*Chl*) assay, 1 g of fresh tissue was ground for 50s in 5 mL tube. After grinding, 3.5 mL 95 % ethanol was added and the mixture was stirred thoroughly and extracted overnight at 4 °C in darkness. The extract was centrifuged at 8000 r/min at 4 °C for 10 min. The supernatant was used for spectrophotometric determination of *Chl a* and *Chl b* at wavelengths of 665 and 649 nm, respectively. The content of these pigments was calculated according to formulas of Lichtenthaler and Wellburn (1983) and expressed as milligrams per kilogram fresh weigh (mg/kg FW).

### 2.3 Phenolic compounds assay

#### 2.3.1 TPC assay

The TPC were assayed based on the methodology of Folin-Ciocalteu procedure (Singleton & Rossi, 1965) and our previous method (Zhan et al., 2025) with slight modification. A 1 g leaves was homogenized for 50s in a 5 mL tube. The homogenate was mixed thoroughly in 3.5 mL 95 % methanol and allowed to stand for 2 h at 4 °C with stirring every 30 min. The homogenate was centrifuged at 8000 r/min for 10 min at 4 °C, and the supernatant was used for assay of the TPC. The assay involved in mixing 20 μL aliquot of the methanol extract with 1 mL of Folin-Ciocalteu reagent. After standing for 3 min at room temperature, 980 μL of 7.0 % sodium carbonate/water (*w*/*v*) was added to the solution. The contents of the tube were thoroughly stirred before incubation at 20 °C for 30 min. The absorbance was read at 760 nm and the result was expressed as milligrams gallic acid equivalents per kilogram fresh weight (mg gallic acid/kg FW).

#### 2.3.2 Phenolic acids and flavonoid compounds assay

For extraction, a quota of 0.1000 g freeze-dried powder was extracted in 10 mL 80 % frozen methanol on shaker for 30 min. The extract was then centrifuged at 6000 r/min for 20 min at 4 °C. The supernatants were collected, and the residue was re-extracted twice under the same conditions. All three supernatants were combined, transferred to a rotary evaporation flask, and concentrated to approximately 2 mL. The concentrated extract was filtrated and purified using a pre-activated C_18_ solid phase extraction (SPE) column (500 mg/6 mL, Tianjin Bona Agela Technology Co., Ltd., Tianjin, China). The elution was collected and evaporated under a stream of nitrogen. The residue was redissolved in methanol to a final volume of 1 mL. After filtrated through a 0.22 μm nylon filter membrane, the filtrate was used for assay of PAs and flavonoid compounds.

The PAs was analyzed using Ultra High Performance Liquid Chromatography and Tandem Mass Spectrometry (UHPLC-MS) (Waters xevo tq-s, Waters Company, America) equipped with a Waters ACQUITY UPLC® BEH C18 column (2.1 mm × 100 mm, 1.7 μm). The mobile phase consisted of three solvents: A, 100 % acetonitrile, B, 100 % methanol, and C, water: formic acid = 100:0.01 (*v*/v). Analysis was performed using a gradient program starting with 5–15 % A, 5–15 % B, and 90–70 % C (0–1.0 min), 15 % A, 15 % B, and 70 % C (1.0–1.5 min), 15–20 % A, 15–20 % B, and 70–60 % C (1.5–2.0 min), 20–30 % A, 20–30 % B, and 60–40 % C (2–5 min), 5 % A, 5 % B, and 90 % C (5.1–7 min). The injection volume was 1.0 μL and the flow rate was 0.3 mL/ min at 40 °C. The MS parameters were set as: solvent removal gas flow rate with 800 L/Hr; electrospray ionization temperature at 500 °C and curtain gas with 7.0 Bar. The scanning mode is negative ion mode. The PA was quantified by comparing the peak area with standard phenolic acids (Weiye Metrology and Technology Research Group Co., Ltd., Xinyang, China).

Flavonoid compounds were analyzed using an Ultra High Performance Liquid Chromatography and Tandem mass spectrometry (UHPLC-MS) (Agilent 1290 Ultivo LC/TQ, Agilent Technology Co., Ltd., America) equipped with an Agilent ZORBAX Eclipse Plus C18 (2.1 mm × 50 mm, 1.8 μm). The mobile phase consisted of two solvents: A, water: formic acid = 100:0.01 (*v*/v), B, acetonitrile. Analysis was performed using a gradient program starting with 90–70 % A, 10–30 % B (0–0.5 min);70–60 % A, 30–40 % B (0.5–1.0 min); 60–40 % A, 40–60 % B (1.0–4.0 min);90 % A, 10 % B (4.1–5.0 min). The injection volume was 2.0 μL and the flow rate was 0.3 mL/min at 35.0 °C. The Flavonoid compounds was quantified by comparing the peak area with standard flavonoid compounds (Weiye Metrology and Technology Research Group Co., Ltd., Xinyang, China).

### 2.4 Total ascorbic acid and ascorbic acid assay

Total ascorbic acid (TAA) and AA were determined spectrophotometrically according to protocol of Kampfenkel et al. (1995). A 1 g fresh frozen tissue was ground in 3.5 mL 6 % freezing trichloroacetic acid solution. The homogenate was centrifuged at 8000 r/min for 20 min at 4 °C and the resultant supernatant was immediately used for TAA and AA analysis. The dehydroascorbic acid (DHA) content was computed from the difference between the TAA and AA. The results were expressed as milligrams per kilogram fresh weight (mg/kg FW) based on standard curves produced by freshly prepared l-ascorbic acid solution.

### 2.5 Total antioxidant capacity assay

The extraction solution was prepared as described in section 2.3.1. TAC was assayed using three methods, namely FRAP (ferric reducing antioxidant power) (Benzie & Strain, 1996), ABTS (2,2-azinobis (3-ethyl-benzothiazoline-6-sulfonic acid) (Re et al., 1999), and DPPH (2,2-diphenyl-1-picrylhydrazyl) (Larrauri et al., 1998), respectively. In FRAP assay, fresh FRAP reagent was prepared by mixing 300 mM sodium acetate-acetic acid buffer (pH 3.6), 20 mM FeCl_3_, and 10 mM TPTZ (2,4,6-tripyridyl-s-stiazine) in 10:1:1 proportion. FRAP analysis was carried out by mixing 50 μL extract and 950 μL fresh FRAP reagent. TAC values were calculated according to standard curve made by the fresh ammonium ferrous sulfate and expressed as millimoles ferrous ion per kilogram fresh weight (mmol Fe^2+^/kg FW).

Fresh ABTS^●+^working solution was prepared by mixing 7 mM ABTS^●+^ and 2.45 mM potassium persulfate in equal quantities (*v*/v) and allowing them to react for 16 h in dark. For each assay, 0.1 mL of the methanol extract was mixed with 1.9 mL of the ABTS^●+^ working solution. The mixture was allowed to stand for 6 min at room temperature before the absorbance was detected at the wavelength of 734 nm. The results were expressed as millimoles of Trolox equivalents per kilogram fresh weight tissue (mmol Trolox/kg FW) based on a standard curve generated using Trolox solution (10–100 μM).

Fresh DPPH working solution was diluted with methanol to the absorbance value of 1.1 ± 0.02 at 515 nm before use. In assay, 0.05 mL extract was mixed with 1.95 mL DPPH solution and reacted at 37 °C for 30 min before the absorbance was recorded at 515 nm. The results were expressed as millimoles of Trolox equivalents per kilogram fresh weight (mmol Trolox/kg FW).

### 2.6 Enzymes activities assay

#### 2.6.1 Antioxidant enzyme activities assay

Fresh tissue (0.5 g) was homogenized in 5 mL of ice-cold 50 mM phosphate buffer solution (PBS, pH 7.8) containing 0.2 mM ethylenediaminetetraacetic acid (EDTA) and 2 % polyvinylpyrrolidone (PVP). The homogenate was centrifuged at 8000 r/min for 20 min at 4 °C. The supernatant was collected as a crude enzyme solution for analysis of SOD, CAT, and POD.

SOD activity was assayed by its ability to inhibit photochemical reduction of nitro blue tetrazolium (NBT) at 560 nm (Beauchamp & Fridovich, 1971). In this assay, 25 μL crude enzyme was mixed with 3 mL NBT react solution that contained 0.75 mM NBT, 130 mM L-methionine, 0.1 mM EDTA·Na_2_, and 0.02 mM riboflavin in 50 mM PBS (pH 7.8). The reaction was started by adding riboflavin and lasted for 20 min under light condition at 25 °C. The absorbance was recorded at 560 nm. One unit (U) of SOD activity was defined as the quantity of enzyme that reduced the absorbance of samples to 50 %. The specific SOD activity was expressed as U/g FW. CAT activity was determined according to Cakmak and Marschner (1992) with slight modification. It was assayed by mixing 0.2 mL extract and 1.6 mL of 25 mM PBS (pH 7.0, containing 0.1 mM EDTA). The reaction was started by adding 0.2 mL 100 mM H_2_O_2_ solution. The absorbance was recorded at the wavelength of 240 nm. One unit (U) of CAT activity was defined as the amount of enzyme that causes a decrease in absorbance of 0.01 per minute and expressed as U/g FW. POD activity was determined according to our previous description (Zhan et al., 2025) with modification. In this assay, the react solution contained 0.2 mL crude extract, 0.1 mL 20 mM H_2_O_2_, 0.1 mL 1 % guaiacol solution, and 1.6 mL 25 mM PBS (pH 7.0, containing 0.1 mM EDTA). The absorption value was read at 470 nm due to guaiacol oxidation. One unit (U) of POD activity was defined as the amount of enzyme that causes an increase in absorbance of per minute and expressed as U/g FW.

#### 2.6.2 Phenolic synthetic enzyme activities assay

For phenolic enzyme analysis, 0.5 g of fresh sample was homogenized in 4 mL of 0.1 M PBS (pH 8.0) containing 4 % PVPP, 2 mM EDTA, and 5 mM β-mercaptoethanol. The homogenate was centrifuged at 12000 *g* at 4 °C for 30 min, and the supernatant was used for enzyme assay. PAL activity was determined according to our previous description (Zhan et al., 2025) with slight modification. Briefly, the reaction mixture consisted of 0.1 mL supernatant and 1.9 mL 50 mM boric acid buffer (pH 8.0) containing 20 mM *L*-phenylalanine. The mixture was incubated for 1 h at 37 °C before the absorbance was measured at 290 nm. One unit (U) of PAL activity was defined as the amount of enzyme to increase absorbance by 0.01 per hour at 290 nm.

*trans*-Cinnamic acid 4-hydroxylase (C4H) activity was conducted as described by Lamb and Rubery (1975) with slightly modification. The assay mixture was made of 0.1 mL 50 mM cinnamic acid, 0.2 mL 0.4 g/L NADPH solution, 1.65 mL 0.1 M PBS (pH 7.6), and 0.05 mL crude enzyme. The reaction is allowed to proceed for 30 min at 30 °C. The absorption was measured at 340 nm against a reference sample lacking cinnamic acid which had been taken through the same procedure. C4H activity is defined as the amount of enzyme that causes an increase in absorbance of 0.01 per minute at 340 nm and expressed as U /g FW. The 4-coumaric acid: CoA ligase (4CL) activity detection was conducted using 4-Coumarate CoA Ligase Assay Kit (Sangon Biotech (Shanghai) Co., Ltd., Shanghai, China). One unit (U) of 4-coumarate: coenzyme A ligase (4CL) activity was defined as the amount of enzyme that catalyzes the formation of 1 nmol of 4-coumaroyl-CoA per hour and expressed as U /g FW.

### 2.7 Headspace gas, fresh weight loss, and dry matter content assay

The content of headspace gas (O_2_ and CO_2_) was measured using MOCON® portable O_2_/CO_2_ analyzer (Mocon Inc., USA). FWL was measured by weighing the samples at each sampling day and calculated progressively based on a comparison of samples weight at storage. DM content was assayed by drying 5 g of fresh leaves in an oven at 65 °C until constant weight.

Unless specified, all homogenization operations were performed using a high throughput tissue grinder (SCENTZ-48, Ningbo Xinzhi Biotechnology Co., Ltd., Ningbo, China). All centrifugation were performed using the refrigerated high-speed centrifuge (3 K30, Sigma Laborzentrifugen GmbH, Osterode, Germany); All colorimetric analyses were conducted using the UV–visible spectrophotometer (JH754PC, Shanghai Jinghua Technology Instrument Co., Ltd., Shanghai, China).

### 2.8 Gene expression of PAL, C4H, and 4CL

Total RNA was extracted using Spin Column Plant Total RNA Purification Kit (Sangon Biotech (Shanghai) Co., Ltd., Shanghai, China). cDNA was synthesized using PrimeScript™ kit (Monad (Suzhou) Biotechnology Co., Ltd., Suzhou, China) according to the manufacturer's instructions. RT-qPCR was performed using the SYBR qPCR Master Mix (Vazyme Biotech Co.,Ltd., Nanjing, China) and gene-specific primers on the QuantStudio 5 Real-Time PCR system (Thermo Fisher Scientific, USA). The RT-qPCR reactions were performed in a final volume of 20 μL including 1.0 μL of template cDNA, 10 μL of Ultra SYBR mixture, 0.4 μL of each primer, and 8.2 μL of RNase-free water. The thermal cycle conditions were as follows: initial denaturation at 95 °C for 30 s, followed by 40 cycles of 95 °C for 10 s, 60 °C for 30 s, and 60 °C for 30 s. Primer sequences were synthesized by Sangon Biotech Co., Ltd., Shanghai, China (Table S1). The specificities of the primers were confirmed through NCBI primer BLAST (https://www.ncbi.nlm.nih.gov/tools/primer-blast/, accessed on 14 December 2024). The relative expression of gene was calculated using the 2-ΔΔCT method (Livak & Schmittgen, 2001).

### 2.9 Statistical analysis

All assays were done at least in triplicate, and results are presented as means ± SD. Data from triplicates for each treatment were submitted to analysis of variance (ANOVA) using Statistical Package for Social Science (Version 26.0) software. One-way ANOVA followed by the Duncan test at 0.05 confidence level was used to identify significant differences. Pearson correlation analyses between variables were conducted via bivariate analysis of the variance model. The Pearson correlation and significance were expressed as correlation coefficient (R) and *p* value, respectively.

## 3 Results

### 3.1 UVA-LED irradiation dose selection

**Fig. 2:**
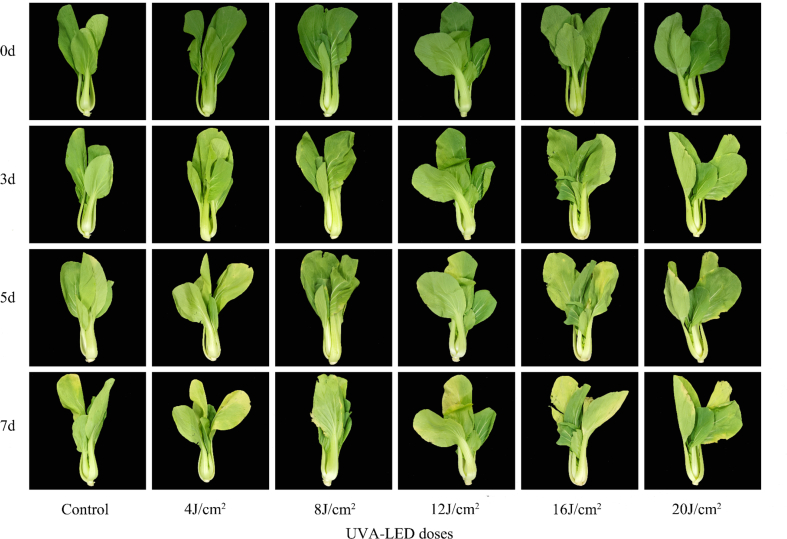
Effects of UVA-LED irradiation doses on the senescence symptoms of minimally processed pakchoi stored at 8 °C for 7 days.

**Fig. 3:**
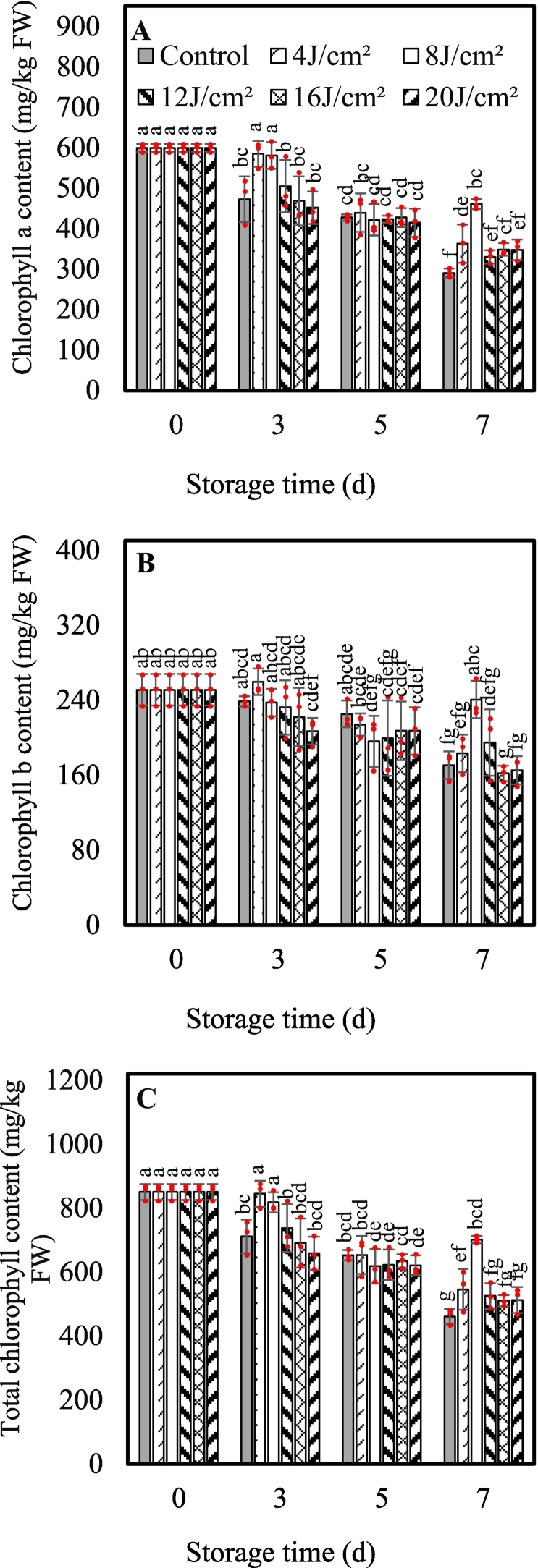
Effects of UVA-LED irradiation on content of chlorophyll of minimally processed pakchoi stored at 8 °C for 7 days. (A, chlorophyll a; B, chlorophyll b; C, total chlorophyll). The symbol “·” represents the individual independent replicates. Error bars represent the standard deviation of three replicates.

UVA-LED irradiation doses largely affected the MPP leaves senescence progress during storage. Apparently, treatment of 8 J/cm^2^ doses delayed tissue yellowing and preserved leaves green color throughout 7d storage (Fig. 2). In detail, MPP leaves irradiated with 8 J/cm^2^ doses were green and fresh at d5, and still marketable at d7 although with a slight edge yellowing symptom. Comparatively, leaves from control and treatments of 4, 12, 16, and 20 J/cm^2^doses showed visible yellowing symptoms at d5 storage; The outer one or two leaves turned more and more yellowish over time, unacceptable for market at d7 storage. Particularly, 16 and 20 J/cm^2^ doses resulted in the leaves edge-burning symptom accompanying small black spots (Fig. 2). Thus, treatment of 8 J/cm^2^ dose was effective to prolong about 2 days shelf-life of MPP. In addition, the change trend of *Chl* content was in line with the senescence symptom development, that is, 8 J/cm^2^ dose treatment preserved significantly more *Chla, Chlb*, and total *Chl* content, being 1.59-, 1.41-, and 1.52-fold of those in control plants at d7, respectively (Fig. 3). Other doses treatments showed statistical same pigments content with control, except that 4 J/cm^2^ dose resulted in significant higher of 24.96 % *Chla* and 18.50 % total *Chl* content than the control at the end of storage (Fig. 3). Thereby, taking overall consideration of yellowing symptom, shelf-life, and *Chl* content, 8 J/cm^2^ dose was chosen and applied in the second experiment to investigate its influences on antioxidant property of MPP during 7d storage.

### 3.2 Effects of UVA-LED irradiation on phenolic compounds

#### 3.2.1 PAs and TPC

**Fig. 4:**
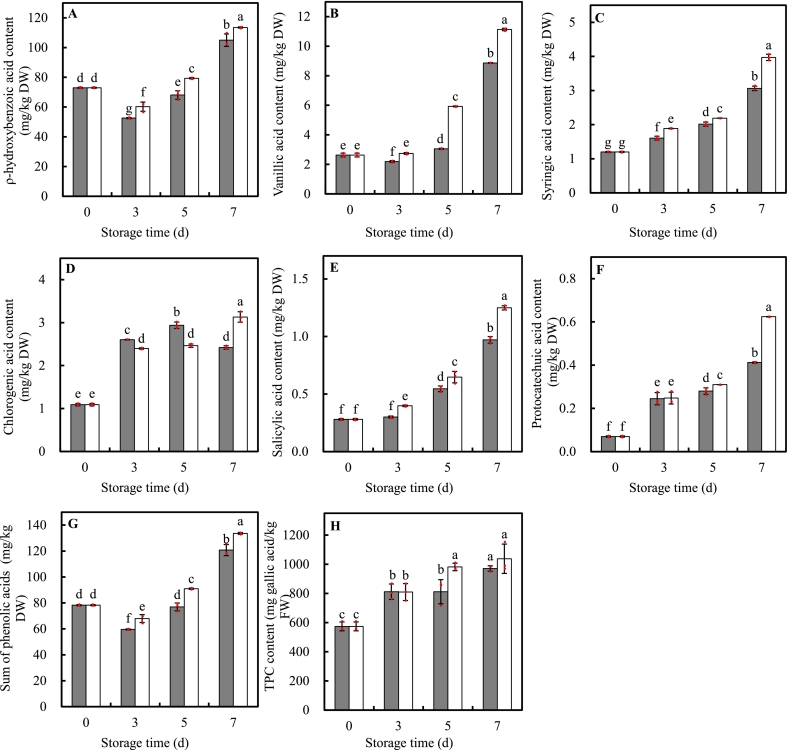
Effects of UVA-LED irradiation on content of phenolic acids (PAs) and total phenolic compounds (TPC) of minimally processed pakchoi stored at 8 °C for 7 days. (■, control; □, UVA-LED). (A, ρ-hydroxybenzoic acid; B, vanillic acid; C, syringic acid; D, chlorogenic acid; E, salicylic acid; F, protocatechuic acid; G, sum of phenolic acids; H, total phenolic compounds). The symbol “·” represents the individual independent replicates. Error bars represent the standard deviation of three replicates.

The composition and quantity of major PAs in MPP were profiled in Fig. 4A-F. Total of six individual PA were detected, including one hydroxycinnamic acid (chlorogenic acid) and five hydroxybenzoic acids (ρ-hydroxybenzoic acid, vanillic acid, syringic acid, salicylic acid, and protocatechuic acid). Among them, ρ-hydroxybenzoic acid represented the dominant composition of phenolic acids in MPP, accounting for 93.26 % of total phenolic acid detected, followed by vanillic acid, syringic acid, chlorogenic acid, and salicylic acid. The proportion of protocatechuic acid was the lowest with an initial value of 0.07 mg/kg DW. The sum of these PAs content was 78.25 mg/kg DW (Fig. 4G).

As expected, all PAs showed similar upward trend, markedly rising without peaks during the whole storage regardless of treatments, with exception of chlorogenic acid that increased and peaked at d5 before declining at d7 in control leaves (Fig. 4A-F). UVA-LED treatment significantly promoted the accumulation of individual PA, resulting in a significantly higher PAs content in treated leaves than the control during the whole storage. At the end of storage, ρ-hydroxybenzoic acid, vanillic acid, syringic acid, chlorogenic acid, salicylic acid, and protocatechuic acid in leaves treated by UVA-LED were 1.08-, 1.26-, 1.30-, 1.29-, 1.29-, and 1.51-fold of their counterparts in control leaves, respectively (Fig. 4A-F). The sum of PAs content in treated leaves were 18.21 % and 10.66 % more than that in control leaves at d5 and d7 storage, respectively (Fig. 4G), implying UVA-LED irradiation triggered de novo synthesis of PAs. Among de novo synthesis PAs induced by UVA-LED, protocatechuic acid showed the largest increase amount with nearly 9.00 times increase respected to the initial value, followed by salicylic acid, vanillic acid, syringic acid, and chlorogenic acid with 4.45-, 4.23-, 3.31-, and 2.87-fold increase, respectively. However, ρ-hydroxybenzoic acid had moderate accumulation, only 1.56 times increases compared to the initial value (Fig. 4A).

The TPC content showed a similar tend to that of PAs, exhibiting a significant increase over time in leaves from both UVA-LED treatment and control (Fig. 4H). Notably, UVA-LED irradiation significantly enhanced TPC accumulation by 1.21-fold compared to the control at d5 storage. However, at d7, there was no statistically significant difference in TPC between treated and control samples. An extremely significant correlation was observed between TPC and the sum of PAs (*R* = 0.641, *p* < 0.01) (Fig. S1), and this strong relationship was consistent between TPC and individual PA, except for chlorogenic acid (Table S2).

#### 3.2.2 Flavonoid compounds

**Fig. 5:**
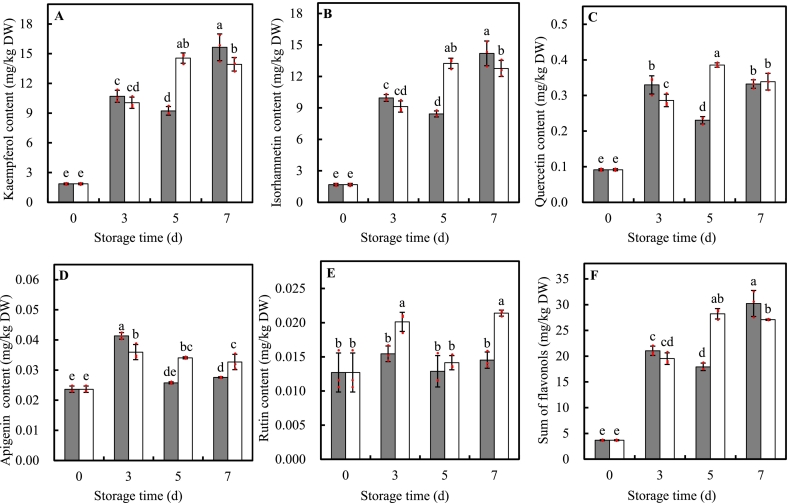
Effects of UVA-LED irradiation on flavonoids compounds content of minimally processed pakchoi stored at 8 °C for 7 days. (■, control; □, UVA-LED). (A, kaempferol; B, isorhamnetin; C, quercetin; D, apigenin; E, rutin; F, sum of flavonols). The symbol “·” represents the individual independent replicates. Error bars represent the standard deviation of three replicates.

Total of five flavonoids substances including kaempferol, isorhamnetin, quercetin, apigenin, and rutin were detected in MPP leaves with considerable variations in concentrations (Fig. 5A-E). Kaempferol and isorhamnetin represented for the prominent individual flavonoids with initial concentration of 1.86 and 1.69 mg/kg DW, accounting for 50.62 % and 45.90 % of sum of all flavonoid compounds detected, respectively (Fig. 5A and B). Quercetin, apigenin, and rutin were in low concentration of less than 0.1 mg/kg DW before storage (Fig. 5C-E). These flavonoid compounds were significantly regulated by UVA-LED over time and changed with different dimensions. The levels of kaempferol, isorhamnetin, and quercetin in UVA-LED treated leaves increased and reached the peaks at d5, being 1.58-, 1.57-, and 1.67-fold of that in control, respectively (Fig. 5A-C). Apigenin content peaked at d3 and declined thereafter in both treated and control leaves; however, UVA-LED treatment delayed this decrease, resulting in 32.16 % and 18.51 % higher apigenin content than the control at d5 and d7 of storage, respectively (Fig. 5D). Rutin content in leaves treated by UVA-LED changed irregularly, that is, it significantly increased at d3 and decrease at d5, thereafter increased again at d7, being 1.30- and 1.47-fold as much as the control at d3 and d7 storage, respectively (Fig. 5E). All in all, UVA-LED irradiation remarkably enhanced 57.50 % more accumulation of flavonoid compounds at d5, but 10.39 % less at d7 storage compared to control, (Fig. 5F).

### 3.3 Effect of UVA-LED on ascorbic acid and TAC

**Fig. 6:**
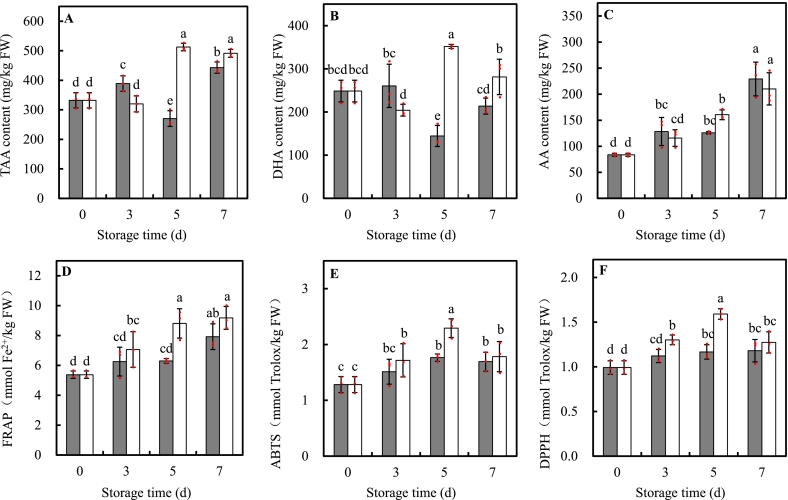
Effects of UVA-LED irradiation on content of ascorbic acid and total antioxidant capacity (TAC) of minimally processed pakchoi stored at 8 °C for 7 days. (■, control; □, UVA-LED). (A, TAA-total ascorbic acid; B, DHA-dehydroascorbic acid; C, AA-ascorbic acid; D, FRAP-ferric ion reducing antioxidant power; E, ABTS-2,2′-azino-bis(3-ethylbenzothiazoline-6-sulfonic acid); F, DPPH-1,1-diphenyl-2-picrylhydrazyl radical). The symbol “·” represents the individual independent replicates. Error bars represent the standard deviation of three replicates.

UVA-LED had significant effect on TAA evolution over time, resulting in 1.90 and 1.11 times of the control at d5 and d7 storage, respectively. Specifically, in leaves treated with UVA-LED, TAA content remained stable during the initial 3d storage, then sharply increased to a peak on d5, showing a 54.27 % rise, and remained stable through d7. In contrast, TAA content in control leaves increased at d3, declined at d5, and rose again by d7, resulting in a 33.30 % increase by the end of storage (Fig. 6A). DHA showed similar trend to TAA, that is, DHA content in leaves treated by UVA-LED were 2.43 and 1.32 times of that from control at d5 and d7 storage, respectively (Fig. 6B). AA content was not significantly affected by UVA-LED and significantly increased over time in both treated and control samples (Fig. 6C).

Three different methods were used to assay the TAC with similar change trends, indicating these methods were feasible and reliable in evaluating pakchoi TAC. TAC values increased over time regardless of treatments, of which UVA-LED resulted in a remarkable elevation in TAC values than control at d5 storage. At d5, TAC values in treated leaves were 30 %–40 % (29.92 % for ABTS, 36.34 % for DPPH, and 39.77 % for FRAP) higher than those in control. While, at d7, TAC values were statistically same between treated and control samples (Fig. 6D-F).

### 3.4 Effect of UVA-LED on enzyme activities

#### 3.4.1 Antioxidant enzymes activities

**Fig. 7:**
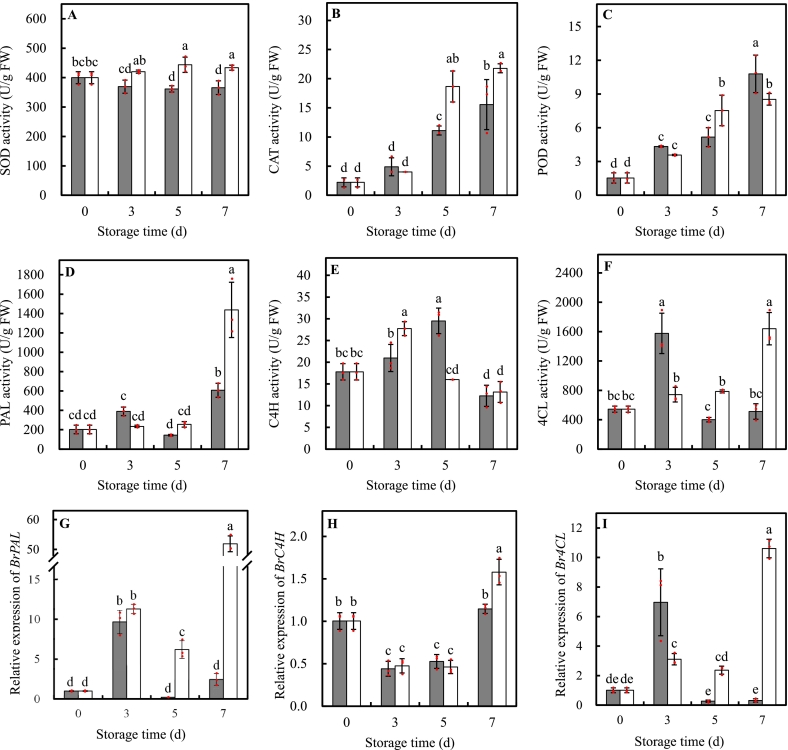
Effects of UVA-LED irradiation on the activities of enzymes and gene expressions in minimally processed pak choi stored at 8 °C for 7 days. (■, control; □, UVA-LED). (A, SOD-superoxide dismutase; B, CAT-catalase; C, POD-peroxidase; D, PAL-phenylalanine ammonialyase; E, C4H-cinnamic acid-4-hydroxylase; F, 4CL-4-coumarate: CoA ligase; G, *BrPAL*, E, *BrC4H*, F, *Br4CL*). The symbol “·” represents the individual independent replicates. Error bars represent the standard deviation of three replicates.

UVA-LED significantly induced the increase in activities of antioxidant enzymes over time, resulting in higher levels of SOD (+ 22.75 %), CAT (+ 68.00 %), and POD (+ 45.81 %) enzyme activities than the control at d5 storage, respectively (Fig. 7A-C). In detail, SOD, POD, and CAT activities in UVA-LED treated leaves significantly increased at d5 and remained constant thereafter, with increases of 1.11-, 4.90-, and 8.40-fold respected to their initial values, respectively. In control leaves, SOD activity significant descended from d5 storage with 8.51 % decrease at the end of storage (Fig. 7A); POD and CAT showed a rapid increase over time without peaks; they displayed an increment in same dimension, being approx 7-fold higher than their initial values at the end of storage (Fig. 7B-C).

#### 3.4.2 PAL, C4H, and 4CL activities and their gene expressions

PAL enzyme activity remained stable and statistically unchanged in both UVA-LED treatment and the control samples during the first 5d of storage. However, by 7d of storage, UVA-LED treatments significantly induced an increase in PAL activity, reaching a level of 2.36 times higher than that of the control (Fig. 7D). C4H activity in both UVA-LED treatment and the control exhibited a similar trend of increasing initially and then declining over the whole storage. In the UVA-LED treated leaves, C4H activity peaked on 3d storage, which was 1.32 times higher than that of the control. While, the control leaves peaked C4H activity at 5d storage, which was 1.84 times higher than that of UVA-LED treatment. By d7 storage, the levels of C4H activity were statistically same in both groups (Fig. 7E). The 4CL activity differed markedly between UVA-LED treatment and the control. In the UVA-LED treatment samples, 4CL activity remained unchanged during 5d storage, then significantly increased to its maximum value on 7d storage. In contrast, 4CL activity in the control samples initially increased, peaked at 3d, and then decreased. Consequently, UVA-LED induced 1.96- and 3.20-fold higher 4CL activity compared to control at 5d and 7d storage, respectively (Fig. 7F).

The relative expressions of *BrPAL*, *BrC4H*, and *Br4CL* significantly affected by UVA-LED irradiation (Fig. 7G-I). Compared to the initial values, UVA-LED treatment upregulated *BrPAL* expression during the whole storage, with increases of 11.28-, 6.19- and 51.81-fold at 3d, 5d, and 7d storage, respectively. In the control samples, *BrPAL* expression increased only at 3d storage, reaching 9.65-times higher than the initial value. As a result, the relative expression levels of *BrPAL* in UVA-LED treated samples were 28.09 times and 21.25 times higher than those in the control samples at d5 and d7 storage, respectively (Fig. 7G). The relative expression of *BrC4H* showed a similar trend in both treated and control samples during storage, decreasing at d3 followed by unchanged level at d5. However, at d7, *BrC4H* expression significantly upregulated by UVA-LED, reaching a level of 1.38-time higher than that of the control (Fig. 7H). *Br4CL* relative expression showed a similar pattern of *BrPAL* during the whole storage. In UVA-LED treated samples, *Br4CL* significantly increased at d3 and d7, reaching 3.08 and 10.52 times higher regarding to the initial value. However, in the control samples, *Br4CL* upregulated only at d3, followed by a decline to level comparable to the initial values. Finally, UVA-LED treatment resulted in 8.69- and 33.33-fold higher *Br4CL* levels than those in the control samples at d5 and d7 storage, respectively (Fig. 7I).

### 3.5 Headspace gas, DM and FWL

Both UVA-LED treatment and control exhibited a similar change trend in headspace gas concentration, that is, a significant decrease in O_2_ content and an increase in CO_2_ content over time (Fig.S2A and B). The level of these gases remained statistically same in both treated and control samples until d5 storage. However, at d7 storage, UVA-LED treatment resulted in a significantly higher O_2_ (+3.15 %) and lower CO_2_ (−8.20 %) level than the control (Fig.S2A and B). Dry matter content was not significantly affected by UVA-LED treatment. In both the treatment and control samples, dry matter remained stable during the first 5 days of storage and significantly increased by day 7, reaching statistically similar levels (Fig. S2C). The FWL, as expected, showed a gradual increment throughout storage regardless of treatments, with UVA-LED treatment accelerating this trend. After 7d storage, the FWL in UVA-LED treated samples was 0.91 %, which was 27.12 % higher than that in control (Fig.S2D).

## 4 Discussion

### 4.1 UVA-LED alleviates the yellowing and extends the shelf-life of MPP, but highly dose-dependent

The degree of plant response to UV light typically depends on the irradiation dose and spectrum range; High dose of UVA inhibited the growth and biomass accumulation of plant by causing disruption on the photosynthesis apparatus; but low dose of UVA reduced the photosynthates such as soluble sugar, protein, and vitamin C (He et al., 2021). In the present study, results from the first trial clearly illustrated the application of UVA-LED irradiation before storage could significantly alleviate the chlorophyll deterioration and yellowing development, thus extending 2 days shelf-life of MPP; but this impact was highly dose-dependent, that is, an 8 J/cm^2^ dose irradiation was the most effective compared to other dose treatments (Fig. 2, Fig. 3), underlining the importance of carefully considering dose in practical applications of UVA-LED for food industry and vertical farming. This finding aligns with the work of Chen et al. (2019) who reported that the stimulating effect of UV-A on plant was not a simple linear dose-response relationship, emphasizing that both insufficient and excessive UVA exposure can be suboptimal or detrimental. Similar dose-dependent responses to UVA irradiation have been observed on Chinese kale (He et al., 2021) and lettuce (Chen et al., 2019) during preharvest cultivation, with the latter exhibiting signs of photoinhibition to high intensity (30 μmol m^−2^ s^−1^) of UVA at 365 nm. However, to date, few studies have addressed the effect of UVA-LED application on postharvest vegetables traits, despite its potential to induce photochemicals and enrich photosynthetic pigments within the safe spectrum (Sonntag et al., 2023), thus no comparative data for comparison. Particularly, researches comparing a dose effect of UVA irradiation on the postharvest plant is scarce, highlighting the innovative nature and importance of the current study in addressing this knowledge gap. As a matter of fact, only in recent years have UVA-LEDs been used as either a part of, or the sole light source for plant growth experiments, due to advances in LED technology that enable precise control both spectrum and intensity (Verdaguer et al., 2017). Brazaityte et al. (2015) found that pre-harvest supplementation of 390 nm UVA-LED could stimulate pakchoi microgreens growth and induces remarkable increases in chlorophyll index. Consistent with this finding, our study confirmed that UVA-LED irradiation at 8 J/cm^2^ dose was favorable to preserve chlorophyll content and green color of MPP leaves during storage. Such positive effect might be attributed to enhancement of photosynthesis by UVA-LED, as the harvested fresh leaves still response to light stimuli and can continue light-dependent biological processes like photosynthesis even during the early stages of storage when exposed under suitable light condition (Zhan et al., 2020). In this study, the peak wavelength of the UVA-LED used was 390 nm (Fig. 1A), very close to visible light spectrum (>400 nm), which is important for plant photosynthesis. Under this circumstance, it is assumed that the pakchoi leaves treated by UVA-LED might continue photosynthesis at initial stage of storage, which possibly contributes to delaying pigment deterioration and tissue senescence. This assumption is strongly supported by the higher O_2_ (+ 3.15 %) and lower CO_2_ (− 8.20 %) concentration observed in packages of UVA-LED treated samples compared to the control (Fig. S2 A and B). These differences in gas concentration were likely resulted from a consumption of CO_2_ and release of the O_2_ during photosynthesis, though the increase of DM content (photosynthates) was not observed in UVA-LED treated samples (Fig. S2 C.). Similar result was also observed by Toledo et al. (2003) who reported that the postharvest light irradiation could effectively maintain the photosynthetic structure and capacity of spinach leaves even the onsets of leaf yellowing, thereby increasing the photosynthesis.

Apparently, at both lower doses (4 J/cm^2^) and higher doses (12 J/cm^2^ and above), UVA-LED irradiation had a negative impact on leaf appearance and chlorophyll content (Fig. 2, Fig. 3). The effect of the lower dose 4 J/cm^2^ can be explained by the insufficient light energy provided, which is likely too minimal to effectively drive photosynthesis in the leaves. On the contrary, the negative effects of higher doses are likely related to the light-induced injury or light inhibition, as excessive UV irradiation is known to result in the generation of free radicals, damaging photosynthetic pigments (Chen et al., 2019; Verdaguer et al., 2017). In the present study, the lower chlorophyll content observed in samples treated with high doses (12 J/ cm^2^ and over) of UVA-LED during the early storage stage (d3) might be explained by light-induced injury or light inhibition mechanisms. However, during the later storage stage (d5), chlorophyll degradation was more likely attributed to the leaf senescence. Similar dose-depended results were also reported by He et al. (2021) who found that a medium dose of UVA-10 (10 W/m^2^) was optimal for producing high-quality Chinese kale leaves, compared to lower dose (UVA-5) and higher dose (UVA-15). The authors stated that the higher dose of UVA (UVA-15) inhibited the biomass accumulation and induced photosynthetic damage, in response, the plants activated the photoprotective mechanisms by reducing chlorophyll a content (He et al., 2021). Additionally, the chlorophyll metabolism is regulated by both the activity of chlorophyll synthetase and the expression of related gene (*BrHEMA1*), as well as several chlorophyll-degrading genes (*BrChlase1, BrChlase2, and BrPPH*) (Zhou et al., 2020). Previous study showed that the white LED light can delay chlorophyll degradation in pak choi leaves by inhibiting activities and expression of degradation-related enzymes and genes (*BrChlase1, BrChlase2, and BrPPH*), while upregulating the expression of the chlorophyll synthetase gene (*BrHEMA1*) (Zhou et al., 2020). Similarly, Xie et al. (2022) illustrated that purple LED light downregulated the expression of chlorophyll degradation - related genes (*BoSGR, BoPAO, BoNYC1* and *BoRCCR*), thereby retained chlorophyll content of broccoli florets. However, in the present study, neither enzyme activities nor gene expression related to chlorophyll metabolism were detected, limiting the understanding of underlying mechanisms driving chlorophyll dynamics in pakchoi under UVA-LED treatments. Further studies are needed to investigate the chlorophyll metabolism network at both physiological and molecular level.

### 4.2 UVA-LED delays senescence through enhancing the enzymatic and non-enzymatic antioxidants of MPP

Postharvest senescence, mainly driven by excessive accumulation of reactive oxygen species (ROS), poses a major challenge for leafy vegetable during supply chain (Xu et al., 2014). This visible change, with yellowing symptom due to the chlorophyll breakdown, can be easily detected by consumers, and thereby directly reduces the consumers perception and limits the shelf-life of fresh produce. Our study illustrated that UVA-LED irradiation at dose of 8 J/cm^2^ significantly alleviated the chlorophyll deterioration and senescence symptoms (Fig. 2, Fig. 3). Such favorable influence might be attributed to the enhancement of antioxidant system by UVA-LED, which comprises enzymatic and non-enzymatic antioxidants to synergistically scavenge ROS and protect cellular homeostasis (Hasanuzzaman et al., 2020). As known, chlorophyll and chlorophyll-degrading enzymes are situated in different localization of chloroplast. Chlorophyll has been found in the thylakoid membrane, while chlorophyll-degrading enzymes has been localized to the inner membrane of the chloroplast envelope. During the plant senescence procedure, the overproduction of ROS leads to the membrane lipid peroxidation, causing the collapse of chloroplast. This may facilitate the physical contact of chlorophyll and its degrading enzymes, thereby accelerating the enzymatic degradation of chlorophyll. Antioxidant enzymes such SOD, CAT, and POD are crucial in preventing oxidative injury through their abilities to scavenge ROS (Xu et al., 2014). In our study, UVA-LED treatment activated the activities of the antioxidant enzymes, with increases of 22.75 % in SOD, 68.00 % in CAT, and 45.81 % in POD at 5 d storage (Fig. 7A-C). These enhancements in antioxidant enzymes activities likely strengthened antioxidant defense system in leaves, facilitating the scavenging of ROS and thereby reducing chloroplast damage and chlorophyll degradation. This was further supported by the positive correlations between activities of these antioxidant enzymes and TAC (Fig. S1). Similarly, Xu et al. (2014) also found blue LED light (470 nm) irradiation stimulates antioxidant activities of SOD, CAT, APX, and POD, thereby inhibiting the lipid peroxidation and postharvest senescence of strawberry.

In addition, nonenzymatic antioxidants such as phenolic compounds, AA, and flavonoids also play a critical role in ROS scavenging, acting in coordination with antioxidant enzymes to creates a high effective antioxidant network to protect plant from oxidative damage and membrane integrity (Hasanuzzaman et al., 2020). As shown in Fig. 4, Fig. 5, Fig. 6, UVA-LED treatment significantly increased the levels of TPC, PAs, flavonoid compounds, and AA, which was also supposed to contribute the improvement of antioxidant system and ROS scavenging ability, thereby alleviating chlorophyll degradation and senescence. This aligned with an early study which demonstrated that purple LED light treatment significantly preserved chlorophyll content and reduced the yellowing of broccoli florets by increasing phenolics contents and maintained the membrane integrity (Xie et al., 2022).

It is worth noting that the TAC values in UVA-LED treated samples were higher on d5 compared to d7 of storage (Fig. 6E-F), with exception of the FRAP method, which showed no statistically significant difference in TAC value between d5 and d7 of storage (Fig. 6D). Such elevated TAC values observed on d5 of storage might be linked to the high levels of enzymatic and non-enzymatic antioxidants, as positive correlations were observed among these antioxidant components and TAC (Fig. S1). However, as shown in Fig. 4, Fig. 5, Fig. 6, Fig. 7, the levels of most antioxidants in samples treated by UVA-LED on d5 were not significantly higher than those on d7 of storage. We speculated the lipophilic antioxidants such as vitamin E and carotenoids, which were not detected in this study, possibly have contributed to the TAC values assayed by the ABTS and DPPH, as both of which are more responsive to lipid-soluble antioxidants. Consequently, the further investigation is necessary to compare the contributions of lipophilic and hydrophilic antioxidant fraction induced by UVA-LED treatment in MPP.

### 4.3 UVA-LED induces the accumulation of phenolic compounds via activating enzymes activity and upregulating their corresponding genes

Applying UV light irradiation to horticultural crops has been considered a simple and effective technology that biofortifies plant to produce a variety of secondary metabolites, including phenols, flavonoids, and anthocyanins (Jacobo-Velázquez et al., 2022). Data on the effects of UV-A radiation on the accumulation of plant metabolites are limited and inconsistent, particularly when compared to the extensive amount of literature on UV-B-induced compound production (Verdaguer et al., 2017). The perception and response of plants to UVA radiation is species-specific and dose dependent, varying from stimulation to inhibition (Brazaityte et al., 2015). For instance, an increase in phenolic compound content in kale was observed under two types (370 nm and 385 nm) of UVA-LEDs irradiation (Lee et al., 2019). Whereas, Brazaityte et al. (2015) revealed that the effect of UVA-LED on phenolic compounds depended on the species and level of exposure, that is, pakchoi microgreens showed an increase in total phenolic compounds at both low (6.2 μmol m^−2^ s^−1^) and high (12.4 μmol m^−2^ s^−1^) irradiation levels, while basil and beet microgreens only exhibited an increase at the high irradiance level. In our study, we found that an 8 J/cm^2^ dose UVA irradiation was effective to induce the accumulation of TPC (1.21-fold), PAs (+18.21 % the sum of PAs), and flavonoids (+57.50 % the sum of flavonoid compounds), and enhance increase of approx. 30–40 % TAC at d5 storage (Fig. 4, Fig. 5, Fig. 6), suggesting UVA-LED irradiation is a feasible strategy to trigger the de novo synthesis of phenolic compounds and improve antioxidant property of MPP leaves when applied with proper dose. This positive effect can be explained by the fact that UVA-LED irradiation upregulated *BrPAL*, *BrC4H*, and *Br4CL* expression, which enhanced the activities of their corresponding enzymes (Fig. 7D-I). Notably, there were extremely positive correlations between *BrPAL* expression and PAL activity (*R* = 0.905*, p* < 0.01), as well as *Br4CL* expression and 4CL activity (*R* = 0.932*, p* < 0.01) (Fig. S1). The elevation in activities of PAL, C4H, and 4CL finally leads to the de novo of synthesis phenolic compounds. This hypothesis was further convinced by the strong and significant correlations observed between the sum of PAs and expression levels of *BrPAL* (*R* = 0.599, *p < 0.01*), *BrC4H* (*R* = 0.848, *p < 0.01*), regardless of treatments (Fig. S1). This is consistent with our previous findings on minimally processed spinach (Zhan et al., 2020), where light irradiation triggered the de novo synthesis of TPC via activating phenylpropanoid pathway. Similar result was also reported by Lee et al. (2019) who found a short-term irradiation of UVA-LED at 375 and 385 nm wavelength promoted an increase in TPC content in kale. The authors attributed such increase to the activation of the transcript level of genes encoding enzymes of PAL, chalcone synthase (CHS), and flavanone-3-hydroxylase (F3H), which, in turn, activated TPC biosynthetic pathways. Additionally, it has been reported that UVA might stimulate the production of phenolic compound by activating the UVA/blue light photoreceptors (Brelsford et al., 2019). There are two best known classes of specific UVA/blue light photoreceptors: crytochromes (CRY1, CRY2, and CRY3) and phototropins (Phot 1, Phot 2) (Brelsford et al., 2019; Briggs & Christie, 2002). In the model plant, CRY1 is involved in photo-morphology (e.g. plant height and phytochemicals). For instance, in *A. thaliana* leaves, the UVA-induced expression of flavonoid biosynthesis genes such as CHS could be initiated via UVA absorption through CRY1 (Fuglevand et al., 1996). The same authors also suggested that the UVA photo-transduction pathway may interact synergistically with UVB-induced pathways to produce transient signals to stimulate CHS promoter function. UVB photoreceptors (e.g. UVR8) could have an impact on UVA-mediated changes in phenolics (Verdaguer et al., 2017). Nonetheless, there is limited information on how UVA independently initiates or interacts with phenolic pathways regulated by other photoreceptors. Additional research is required to mechanistically understand the possible interactions between light photoreceptor and UVA irradiation in term of metabolite accumulation in plants (Verdaguer et al., 2017).

In many plant tissues, two primary classes of phenolic compounds – hydroxycinnamic acids and flavonoids - are involved in epidermal UVA screening (Berli et al., 2010). Hydroxycinnamic acids, including p-Coumaric acid, ferulic acid, chlorogenic acid, and caffeic acid among others, have absorption peak at wavelengths between 310 and 332 nm, and flavonoids absorb most effectively at wavelengths between 350 and 390 nm, respectively (Berli et al., 2010). Thus, the absorption range of these compounds complementarily overlap the UVA spectrum, providing plants a protection from UVA radiation by converting short-wave, high-energy, and highly destructive radiation into longer-wavelength light, which is less harmful to the cellular structures of the leaf (Bilger et al., 2001). In this study, five types of flavonoids compounds (kaempferol, isorhamnetin, quercetin, apigenin, and rutin) were detected, all of which were induced and showed a significant accumulation in at 5d storage in samples treated by UVA-LED (Fig. 5). Compared to flavonoids, only one hydroxycinnamic acid (chlorogenic acid) was detected, with chlorogenic acid being inhibited before d5, and induced at d7 by UVA-LED compared to control (Fig. 4D). Such differences in these compounds might be explained by the fact that the longer wavelength (peak at 390 nm) of UVA-LED used in this study (Fig. 1A), which is much close to the waveband for maxima absorption of most flavonoids. This explanation corroborated by findings showing that UVA irradiation at 385 nm resulted in the higher concentration of flavonoids (quercetin and kaempferol glycosides) in broccoli compared to irradiation at 365 nm (Neugart & Schreiner, 2018; Rechner et al., 2016). However, Lee et al. (2019) illustrated that UVA-LED at 385 nm treatment was more effective in enhancing hydroxycinnamic acid (caffeic acid) content in kale plant than was the UVA-LED at 370 nm treatment. These inconsistent results suggested that there was complicated response pattern and effect of structure-depended reaction on the biosynthesis of phenolic compounds to UV light (Neugart et al., 2012). Each type of phenolic compounds may change depending on the chemical structure, UV wavelength, irradiation dose, and individual plant species. Therefore, multiple factors should be considered, especially UV wavelength and the plant species, when applicating UV irradiation technology to target the desirable polyphenolic compounds.

Last but not least, this study demonstrated UVA-LED treatment significantly accelerated FWL of MPP during storage, resulting in a 27.12 % higher FWL than the control after 7d storage (Fig. S2D). In spite of this, the largest FWL observed of samples treated by UVA-LED was only 0.91 % after 7d storage, being far below the 4–5 % threshold that renders the leafy vegetable texture unacceptable (Nunes & Emond, 2007). This indicated all leaves remained fresh and marketable at the end of storage. It is well known that the FWL of postharvest fresh fruits and vegetables mainly results from respiration and transpiration. Respiration releases water and carbon dioxide via oxidation of respiratory substrates - typically soluble sugar, resulting in a reduction in dry matter content. However, in our study, there was no significant difference in dry matter content between UVA-LED treated samples and the control (Fig. S2C), suggesting FWL was more likely resulted from the transpiration, a process tightly regulated by stomata action. The stomatal aperture accounts for 95 % of water loss from plants (Kozuki et al., 2015). Earlier studies have showed that UVA and blue light induce stomatal opening, mediated by blue light receptors plot 1 and plot 2 (Briggs & Christie, 2002). Therefore, in this study, it is plausible that elevated FWL observed in UVA-LED treated samples was attributed to light-induced stomatal aperture, which increased transpiration fluxes and ultimately leaded to greater fresh weight losses. Similar observations have been reported on lettuce, where light irradiation elevated the FWL by stimulating the stomatal aperture, and the number of stomatal apertures positively correlated to FWL (Martínez-Sánchez et al., 2011). To alleviate such undesirable effects, commercially application of UVA-LED could be integrated with other preservation techniques, such as modified atmosphere packaging, to reduce transpirational water loss. Additionally, maintaining a consistent cold chain and ensuring high relative humidity during long-term storage, transport, and distribution are essential for minimizing FWL of fresh produce throughout the supply chain.

## 5 Conclusion

This study clearly demonstrated that UVA-LED irradiation positively affected the postharvest senescence and enhanced the antioxidant property of MPP, but these effects were highly dose-dependent. Among the tested doses, 8 J/cm^2^ was the most effective. UVA-LED enhanced the antioxidant property of MPP, which was associated with the elevated levels of enzymatic and non-enzymatic antioxidants. Moreover, UVA-LED treatment induced the de novo synthesis of PAs and flavonoid compounds. This was achieved via activation enzymes of PAL, C4H, and 4CL, and upregulation of their corresponding genes. Therefore, UVA-LED irradiation represents a feasible and innovative strategy for postharvest preservation of fresh vegetables, contributing to shelf-life extension and enhancement of antioxidant property. It is interesting that this approach not only offers practical benefits for fresh supply chain, but also presents a novel opportunity for consumer level application. The use of UVA-LED chambers for residential use could offer consumers a simple, chemical free method to treat vegetables at home, potentially enhancing their nutraceutical content prior to consumption, though such devices currently are not yet widely available on the market. Thus, the future research would evaluate the scalability and effectiveness of UVA-LED treatment under both commercial scale and residential use storage conditions.

## CRediT authorship contribution statement

**Yamin Fan:** Writing – original draft, Visualization, Validation, Software, Methodology, Investigation, Formal analysis, Data curation. **Wen Huang:** Writing – original draft, Validation, Supervision, Resources, Methodology, Investigation, Formal analysis, Data curation, Conceptualization. **Han Gao:** Writing – original draft, Validation, Software, Resources, Methodology, Investigation, Formal analysis, Data curation. **Shun Zhang:** Validation, Methodology, Investigation, Formal analysis, Data curation. **Xiaoyan Zhang:** Visualization, Software, Methodology, Data curation. **Jianshe Zhao:** Writing – review & editing, Visualization, Supervision, Project administration, Funding acquisition, Conceptualization. **Lijuan Zhan:** Writing – review & editing, Visualization, Supervision, Project administration, Funding acquisition, Conceptualization.

## Declaration of competing interest

The authors declare that they have no known competing financial interests or personal relationships that could have appeared to influence the work reported in this paper.

## Data Availability

Data will be made available on request.

## Appendix A Supplementary data

Supplementary material 1 Image 1

Supplementary material 2 Image 2
