# Supplementary material for: A novel strategy of using ultraviolet a light emitting diodes (UVA-LED) irradiation extends the shelf-life and enhances the antioxidant property of minimally processed pakchoi (Brassica rapa subsp. chinensis)
Source: Food Chem X. 2025 Jul 22;29:102826. doi: 10.1016/j.fochx.2025.102826 (PMC12318348; doi:10.1016/j.fochx.2025.102826)
Supplement: Supplementary file 1 — Supplementary material 1 [file mmc1.docx]

**Supplementary Materials**

**Fig. S1** The Pearson’s correlations analysis among select variables. The total number of 24 replicates for each parameter. The correlation coefficients are proportional to the color intensity. Positive correlation is displayed in red and negative in blue color. **The correlation is significant at the 0.01 level (two-tailed). ns, not significant.

(TPC-total phenolic compounds; TAA-total ascorbic acid; AA-ascorbic acid; DHA-dehydroascorbic acid; FRAP- ferric ion reducing antioxidant power; ABTS-2,2'-azino-bis (3-ethylbenzothiazoline-6-sulfonic acid); DPPH-1,1-diphenyl-2-picrylhydrazyl radical; SOD-superoxide dismutase; CAT-catalase; POD-peroxidase; PAL-phenylalanine ammonialyase; C4H-cinnamic acid-4-hydroxylase; 4CL-4-coumarate:CoA ligase.)

**Fig. S2** Effects of UVA-LED irradiation on content of O2% (A), CO2% (B), dry matter content(C), and fresh weight loss (D) of minimally processed pakchoi stored at 8 ºC for 7 days. (■, control; □, UVA-LED). The symbol “·” represents the individual independent replicates. Error bars represent the standard deviation of three replicates.

**Table S1** RT-qPCR primer sequence

| **Gene ID** | **Gene name** | **Primer (5' to 3')** | Anneal temperature | Amplification efficiency (%) |
| --- | --- | --- | --- | --- |
| XM_009127096.3 | *BrACTIN* | Forward: ATGTATGTCGCTATTCAGGCTGTTC  Reverse: AGATCGGCACGGTGTGAGAC | 60℃ | 97.23 |
| XM_033281480.1 | *BrC4H* | Forward: CACGATTGGTAGGATGGTACAGAAC  Reverse: ACGATGGTGGAGTGGTGAAGG | 60℃ | 94.92 |
| XM_009146642.3 | *Br4CL* | Forward: CAGAAACAGAGCAGCAACAACAAC  Reverse: TTGGAAGATGTAGTCGTGGAGAGG | 60℃ | 96.84 |
| XM_018584638.2 | *BrPAL* | Forward: TGGATGAAGTGAAGAGAATGGTTATGG  Reverse: ACGGTCGAGATCGCTGCTAC | 60℃ | 96.45 |

**Table S2** The Pearson’s correlations analysis among total phenolic compounds (TPC) and individual phenolic acids. The total number of 24 replicates for each parameter.

|  | **TPC** | **P-hydroxybenzoic acid** | **Vanillic acid** | **Syringic acid** | **Chlorogenic acid** | **Salicylic acid** | **Protocatechuic acid** | **Sum of phenolic acids** |
| --- | --- | --- | --- | --- | --- | --- | --- | --- |
| **TPC** | 1 |  |  |  |  |  |  |  |
| **P-hydroxybenzoic acid** | 0.591** | 1 |  |  |  |  |  |  |
| **Vanillic acid** | 0.755** | 0.962** | 1 |  |  |  |  |  |
| **Syringic acid** | 0.792** | 0.882** | 0.955** | 1 |  |  |  |  |
| **Chlorogenic acid** | 0.726** | 0.253 | 0.433 | 0.642** | 1 |  |  |  |
| **Salicylic acid** | 0.775** | 0.936** | 0.978** | 0.984** | 0.563** | 1 |  |  |
| **Protocatechuic acid** | 0.838** | 0.775** | 0.894** | 0.975** | 0.763** | 0.939** | 1 |  |
| **Sum of phenolic acids acids** | 0.641** | 0.997** | 0.978** | 0.914** | 0.321 | 0.960** | 0.820** | 1 |
| **Note:** (**, Correlation is significant at the 0.01 level; *, Correlation is significant at the 0.05 level) | | | | | | | | |
