# Supplementary material for: A novel strategy of using ultraviolet a light emitting diodes (UVA-LED) irradiation extends the shelf-life and enhances the antioxidant property of minimally processed pakchoi (Brassica rapa subsp. chinensis)
Source: Food Chem X. 2025 Jul 22;29:102826. doi: 10.1016/j.fochx.2025.102826 (PMC12318348; doi:10.1016/j.fochx.2025.102826)
Supplement: Supplementary file 2 — Supplementary material 2 [file mmc2.docx]

**Figure. 1-1**

The mass spectrum of phenolic acids (standards). (A, ρ-hydroxybenzoic acid; B, vanillic acid; C, syringic acid; D, chlorogenic acid; E, salicylic acid; F, protocatechuic acid).

**Figure 1-2**

The mass spectrum of phenolic acids(sample). (A, ρ-hydroxybenzoic acid; B, vanillic acid; C, syringic acid; D, chlorogenic acid; E, salicylic acid; F, protocatechuic acid).

**Figure 2-1** The mass spectrum of flavonoid compounds (standards) (A, kaempferol; B, isorhamnetin; C, quercetin; D, apigenin; E, rutin).

**Figure 2-2** The mass spectrum of flavonoid compounds (sample) (A, kaempferol; B, isorhamnetin; C, quercetin; D, apigenin; E, rutin).
